# Uncovering the Mysteries of Langerhans Cells, Inflammatory Dendritic Epidermal Cells, and Monocyte-Derived Langerhans Cell-Like Cells in the Epidermis

**DOI:** 10.3389/fimmu.2018.01768

**Published:** 2018-07-30

**Authors:** Masayuki Otsuka, Gyohei Egawa, Kenji Kabashima

**Affiliations:** ^1^Department of Dermatology, Graduate School of Medicine, Kyoto, Japan; ^2^Singapore Immunology Network (SIgN), Singapore, Singapore; ^3^Institute of Medical Biology (IMB), Agency for Science, Technology and Research (A*STAR), Biopolis, Singapore, Singapore

**Keywords:** Langerhans cells, inflammatory dendritic epidermal cells, skin inflammation, resident macrophages, dendritic cells

## Abstract

The identity of Langerhans cells (LCs) has been called into question of late due to the increasing evidence that LCs originate from macrophage lineage instead of dendritic cell (DC) lineage as previously thought. For many years, LCs have been assumed to be DCs due to its migratory capabilities. However, recent studies have demonstrated that LCs are from macrophage lineage of the adult fetal liver (FL) progenitor. *Bona fide* LCs are now considered tissue-resident macrophages as they originate from the FL as shown by fate mapping models. In recent years, studies have shown that there are three types of antigen-presenting cells present in the epidermis, such as LCs, monocyte-derived LC-like cells, and inflammatory dendritic epidermal cells (IDECs). Of these, LC-like cells have been characterized in both human and mouse studies, while IDECs have only been described in human studies. This has shed a new light on the area of epidermal macrophages, suggesting that there might be a misidentification and misclassification of LCs. IDECs and LC-like cells have been shown to be present in both steady state and inflammatory state, but they are present in more significant amounts under inflammatory conditions such as atopic dermatitis, ultra violet injury, and psoriasis. In this review, we discuss what is already known and discuss the possible roles of LCs, LC-like cells, and IDECs during inflammation. Most intriguingly, we discuss the possibility of LCs having a dual identity as both a macrophage and a DC. This is shown as LCs are the only tissue-resident macrophage to have shown migratory property-like DCs.

## Introduction of Langerhans Cells (LCs)

Langerhans cells are the only professional antigen-presenting cells that reside in the epidermis under steady-state conditions. They were first described by Paul Langerhans in 1868 as being part of the peripheral nervous system (1); however, recent studies have firmly placed LCs within the hematopoietic system (2–4). In the murine epidermal sheet, several cell populations are dendritic in shape, such as dendritic epidermal T cells (DETCs) and epidermal lymphoid cells (ELCs) (5). Although LCs have a similar shape, they are distinguished by the presence of birbeck granules in their cytoplasm (6). In recent years, the ontogeny of murine LCs has been studied and evidence has suggested that LCs are tissue-resident macrophages in the epidermal sheet. This is surprising as LCs have been shown to have dendritic cell (DC)-like properties such as migrating into the lymph node and the ability to prime T cells (7) leading to the assumption that LCs were part of the DC lineage. The migratory ability of LC is currently of interest and in debate as LCs are the only resident tissue-specific macrophages that have the ability to migrate into the lymph node. Thus, LCs are of special interest in the field of dermatology as their role in inflammation and infection of the skin is poorly understood.

## Development of LC

**Figure 1:**
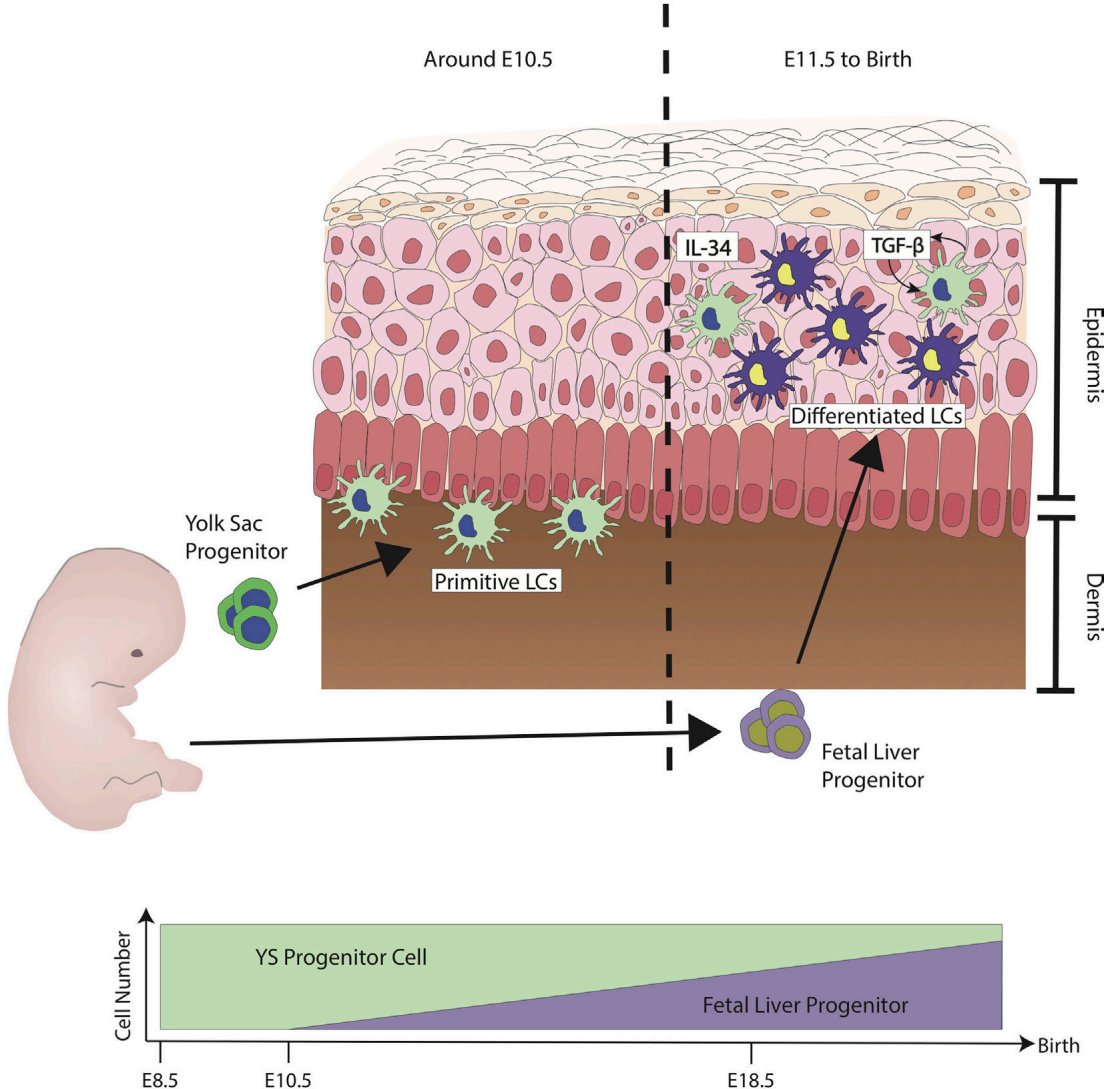
Ontogeny of Langerhans cells (LCs) during steady state. The first wave of LCs that reside in the skin are from the yolk sac (YS) progenitors (green). They populate the skin but are unable to mature fully. At embryonic stage, LCs are immature due to the lack of signals from keratinocytes such as interleukin-34 (IL-34) and transforming growth factor-β (TGF-β). LCs have shown to have the capability to produce TGF-β. TGF-β derived from LCs acts directly on LCs through the autocrine/paracrine signaling pathway. At around E11.5, fetal liver progenitors (purple) start to populate the skin and, similar to the YS progenitors, they are still immature and sparsely distributed. Differentiation into LC has been observed from E18.5 onward, at the same time when keratinocytes start to fully differentiate. Upon birth, LCs have been observed to proliferate to form LC network. During steady state, LCs maintain their network by the low level of proliferation without much contribution from monocytes derived from the bone marrow. Figure adapted from Ginhoux et al. (13).

During ontogenesis, primitive myeloid cells from the yolk sac (YS) seeds the skin and other tissues at around embryonic day (E) 7.5 by a process known as primitive hematopoiesis, through which the first wave of myeloid cells is defined (8). This first wave of cells is from the posterior plate mesoderm in the blood islands of extra-embryonic YS. At around E8.0, a second wave of hematopoietic progenitors called erytho-myeloid precursors (EMPs), which arise from the YS seed the skin. These EMPs are Myb transcription dependent, unlike the first wave, and this second wave of seeding is termed transient definitive hematopoiesis (9). From E8.5 onward, as the blood circulation is established, EMPs populate the fetal liver (FL) (10). During this time, FL-derived progenitors circulate into various tissues including the skin (11). LCs derived from primitive wave are diluted by FL progenitors, and FL derived LCs reside, expand, and self-maintain throughout adult life (12) (refer to Figure 1) (13, 14). LCs express definitive transcriptional factors *Runx3* and *Ahr* (15, 16) only at postnatal development (PND) day 2 (17). From PND day 2 onward, LCs go through a rapid expansion of about a 10- to 20-fold increase in population. They begin to express definitive cellular markers MHC class II and the C-type lectin receptor Langerin (CD207), and in 3 weeks, the adult LC network is established (18, 19). The differentiation of FL progenitors to LCs is highly dependent on the signaling pathway of cytokine transforming growth factor-β (TGF-β), this has been shown in studies where ablation of LCs was observed in TGF-β transcriptional factor *Id2* and Runx3-deficient mice (15, 20). Cytokine interleukin-34 (IL-34) is recognized by colony-stimulating 1 factor receptor (M-CSFR), and M-CSFR is another important cytokine required for the full differentiation to LCs (21, 22). Keratinocytes on the epidermal layer have been shown to express both TGF-β and IL-34, which support the differentiation into LCs, although LCs themselves have shown to have the ability to produce TGF-β. The TGF-β derived from LCs acts directly on LCs through the autocrine/paracrine signaling pathway and is required to facilitate their development and survival (23). Keratinocytes have been shown to play a pivotal role in controlling the position of LCs in different regions of the epidermis by the differential expression of α_v_β_6_ and α_v_β_8_ (24). Once the LC network is formed, LCs self-renew through life without the need of contribution from hematopoietic stem cell (HSC)-derived cell. Unlike most myeloid cells, LCs are radio-resistant and will not be ablated by irradiation (25).

At steady state, a small percentage of LCs have been shown to migrate into skin-draining lymph nodes (dLNs). Other than the role of maturation of LCs, TGF-β has shown to inhibit LCs’ migratory properties, and therefore, its availability determines LC homeostasis (26, 27). In order to maintain LC homeostasis, the conversion of LAP-TGF-β into soluble TGF-β by keratinocyte integrin α_v_β_6_ and α_v_β_8_ is required (24). To sustain its network, LCs replenish themselves through a constant, low-level of proliferation, which is similar to other types of tissue-resident macrophages (19, 28). However, unlike many other cell types, tissue-resident macrophages including LCs proliferate as a differentiated state, which is surprising given that the maintenance is controlled by the same self-renewal gene network (29). The key difference between stem cells and tissue-resident macrophages is that resident macrophages access its self-renewal gene network through macrophage-specific Maf transcriptional factors. Notably, stem cell progenitors are not required for LC network maintenance throughout life.

## LC During Inflammation

Many studies have been conducted to investigate the role of LCs during skin inflammation. The exposure to haptens and ultra violet (UV) light has long been the most popular way to study LC migration and activation. When LCs are activated, they have been shown to have a reduced expression of E-cadherin. E-cadherin is also expressed on keratinocytes and the downregulation of E-cadherin by LCs resembles the disengagement from keratinocytes for migration to the lymph node (30). The migration of LCs into the lymph node has shown to be an important mechanism for the induction of regulatory T cells (Treg). In this study, it has been demonstrated that UV exposure causes immunosuppression by the notable migration of LCs into the lymph node to induce antigen-specific Tregs following UV exposure (31). However, a contrasting study has shown that LCs were not required for UV-induced immunosuppression. Instead, dermal langerin^+^ cells are responsible for the expansion of CD8 T cells, while the UV-induced migration of Treg into the lymph nodes was responsible for the inhibition of contact hypersensitivity (CHS) responses (32–34). The contrasting result is due to the difference in the methodology used. In the study conducted by Schwarz et al., they focused on the suppressive role of LCs during the sensitization phase, while the study by Wang et al. was referring to the suppression of the induction of CHS by UVR (31, 32).

To investigate the role of LCs during inflammation, researchers primarily used LC-deficient mouse models such as huLangerin-diphtheria toxin A (DTA) (langerin-positive cells were knocked out by the expression of a DTA via a human promoter) and muLangerin-diphtheria toxin receptor (DTR) (langerin-positive cells express DTR and were depleted by injecting diphtheria toxin i.p.). These led to the conflicting results on the role of LCs during hapten-induced contact CHS. It was also shown that during the sensitization phase, LCs and langerin^+^ dermal DCs play similar roles by presenting antigens to naive T cells to support their expansion and polarization (35, 36). This is further supported by Zahner et al. who demonstrated this by generating a Langerin-Cre TGF-βR1 mouse model, which has a permanent LC deficiency, and showed a more tampered down CHS response as compared to WT. In Zahner et al.’s study, the regulatory role of LC was not demonstrated (37). When the role of LCs was investigated using the huLangerin-DTA/DTR ablation model, it was shown that LCs play an active role in suppressing immune responses. Conversely, in the muLangerin DTR model, not only did LCs not play a role in immunosuppression, they exacerbated the immune response to allergens (38, 39). In response to the conflicting results, human promoter-driven langerin-DTR mice (huLangerin-DTR) were created to investigate if the conflicting results were due to the method of ablation (inducible vs. constitutive ablation). By using huLangerin-DTR mice, the author was able to ablate only LCs and not langerin-expressing dermal DCs, thereby demonstrating that LCs were indeed responsible for the suppression of immune reactions (40). These studies have shown that differing mouse models can lead to LCs taking on either inflammatory or anti-inflammatory roles. Further studies will be required to investigate the difference between the models and their impacts on LC function.

## Controversy with LC

During skin inflammation, LCs are shown to be affected by having altered proliferation, maturation and migration rates. Migration during inflammation leads to a slight depletion of epidermal LCs, and this loss is compensated by the recruitment of bone-marrow-derived cells into the epidermis. Some monocytes have been shown to be capable of acquiring an LC-like phenotype (41, 42), and this has led to the hypothesis that during skin inflammation, LCs comprised cells with two distinct origins: resident LCs, which originate from the FL, and LC-like cells, which originate from the bone marrow and are only prominent in skin lesions. This has made elucidating the role of *bona fide* resident LCs very challenging.

In mice, the concept of two distinct populations of LCs has been shown by using an *Id2*-deficient mice. TGF-β1 induces the expression of *Id2*, which is essential for the development of LC network in the skin (20). *Id2*-deficient mice lack the LC network from birth, and reconstitution with wild-type mice bone marrow has shown that monocytes are able to differentiate into LC-like cells, supporting the presence of monocyte-derived LC-like cells (43). Skin inflammation causes GR-1^high^ monocytes to be recruited into the skin and develop into LC-like cells (41). As described by Seré et al., recruited LC-like cells are termed as short-term LCs that are derived from Gr-1^high^ monocytes in inflammatory conditions, LC-like cells do not require TGF-β1 signaling, unlike *bona fide* resident LCs. These moLCs are deemed short term as they do not reside in the skin for a long time as compared to resident LCs. They are recruited into the epidermis to replenish the loss of resident LCs during inflammation and replaced by resident LCs once inflammation has stopped (43). In the same study, it was shown that there are two waves of LC recruitment during inflammation. The first wave, termed the inflammatory wave, consists of short-term LC, which are independent of *Id2*, while the second wave, termed the steady-state wave, are long-term LCs, which are dependent on *Id2*. These highlight the existence of two types of LCs under inflammatory conditions. The differentiation of monocytes into LCs occurs 1 week after the induction of mild skin inflammation. GR-1^hi^ monocyte develops into short-term LCs in an *Id2*-independent manner. In contrast, long-term LCs are derived from LC precursors and require *Id2*. These findings show the plasticity in the development of DC and reveal various ways in which LCs develop in steady-state as well as inflammatory conditions (43).

To add to the complexity of LCs, a human study conducted by Martínez-Cingolani et al. has shown that BDCA-1^+^ peripheral blood monocytes of humans are the precursors to human LC-like cells. The differentiation of BDCA-1^+^ DCs into human LC-like cells is driven by the expression of thymic stroma lymphopoietin (TSLP) and TGF-β, which are produced by keratinocytes in lesional skin of atopic dermatitis patients (44, 45). Interestingly, in her study, human LC-like cells have lower expressions of E-cadherin compared to resident *bona fide* LCs (44). This could suggest that these human LC-like cells readily migrate into the dLNs and have very little interaction with keratinocytes as compared to *bona fide* LCs (30). Since her study was conducted in an in vitro manner, she could only suggest that in an inflammatory state, BDCA-1^+^ blood DC are precursors to human LC-like cells. Further studies will be required to determine if these cells are similar to murine LC-like cells from Seré et al.’s study.

In the human study, the identification of another type of professional antigen-presenting cells has also been described; these recruited myeloid cells are termed inflammatory dendritic epidermal cells (IDECs) (46). The identification of IDECs was first described by Wollenberg et al. who observed that there were two distinct types of LCs in lesional skin of atopic eczema and other skin inflammatory diseases. He first identified the CD1a-positive population and then segregated the population by their FCεRI expression (47). The distribution of LC and IDECs in the skin is distinct, IDECs reside in the lower part of the epidermis, while *bona fide* resident LCs reside in the upper layer of the epidermis. The difference in location indicates a functional difference between IDECs and resident LCs. Due to the strategic location of LCs in the upper epidermal layer of the skin, they are able to capture antigens, which are located outside the tight junction (TJ) barrier by extending their dendrites through the TJ barrier (48). In contrast, IDECs residing in the lower layer of the epidermis are unable to capture antigens that are located on the surface of the skin because their dendrites extend horizontally (46).

**Figure 2:**
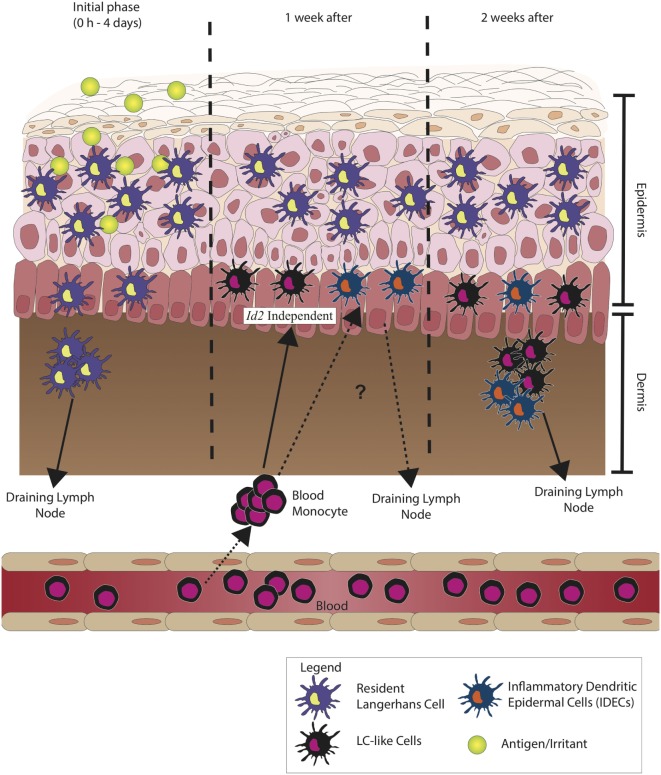
Langerhans cells (LCs) during inflammation. During the initial phase of inflammation, LCs migrate to the draining lymph node, but unlike conventional/classical dendritic cells (cDCs), LCs are observed to peak in number at day 4 after initial sensitization to antigen. The migration of LCs has been observed to be much slower. After 1 week, it is believed that inflammatory dendritic epidermal cells (IDECs) increase in number. These circulating blood monocytes from the bone marrow differentiate to LC-like cells and do not depend on transforming growth factor-β (TGF-β) signaling pathway but are morphologically and phenotypically very similar to resident LCs. IDECs reside in the epidermal–dermal junction, and after 1 week of recruitment, they most likely migrate into the draining lymph node. These show the probable functional difference between LCs and IDECs as the kinetics of recruitment and migration between the two cells is different.

Figure 2 illustrates the current hypothesis of the sequence of events, which occur when self-antigen/antigen initiates an immune response in a human model.

## The Dual Identity of LCs

The dual identity of LCs as a macrophage and DC is a very intriguing phenomenon. The concept of macrophages being able to migrate into the lymph node has baffled immunologists and dermatologists alike for years. Recently, a study has been conducted by Wu et al., using *Mafb-*driven cre transgenic mice to identify whether DCs marked by the expression of *Zbtb46*-green fluorescent protein (GFP) express *Mafb* (49). *Mafb* is a transcriptional factor that is expressed specifically in the myeloid lineage of the hematopoietic system. Its upregulation is observed in differentiation from multipotent progenitors (MPP) to macrophages hence used to distinguish macrophages from other myeloid cells (50). In contrast, *Zbtb46* has been observed to be expressed in all classical DCs; therefore, *Zbtb46* has been used to distinguish classical DCs from all other types of immune cells (51, 52). However, Wu and his colleagues have found that only resident LCs from the epidermis express both *Zbtb46* and *Mafb* simultaneously (49). This makes LCs a unique type of resident macrophage as it expresses both a DC and a macrophage-specific transcription factor. The mechanism of how LCs acquire DC transcriptional factor has yet to be fully understood and whether this population has a contamination with IDECs or LC-like cells has not yet been answered. The recent research conducted by Seré et al. and Martinez-Cingonali et al. hints that *bona fide* LC might have been mischaracterized during inflammation. Whether LCs indeed have a dual identity or have merely been mistakenly defined due to our limited knowledge of the plasticity of circulating monocytes into LC-like cells or IDECs have yet to be clarified.

## Migratory Capability of LC

The migratory capability of LCs is tightly regulated by cytokines. IL-1β, IL-18, and tumor necrosis factor α (TNF-α) have been shown to control the migration of LCs out of the epidermis, and these cytokines are produced specifically by keratinocytes (53–55). It has also been shown that mice deficient in CCR7 have severe defects in LC migration to the skin-dLN, although this defect does not completely inhibit the migration of DC into lymph node (56–58). This suggests that there are CCR7-independent mechanisms, which allow the migration of DCs into lymph nodes.

Langerhans cell migration has been shown to be a two-step process, and the migration of LC into the dermis seems to be highly dependent on CXCR4-CXCL12 manner. CXCR4 has been shown to play a critical role in the maturation and migration of DCs into the lymph node (59). The blocking of CXCR4 has been shown to impair the immune response to antigen-specific CHS. This demonstrates the importance of chemokine CXCR4 in the role of effective cutaneous immune responses during CHS (59). During skin inflammation, the expression of TNF-α induces the expression of CXCL12 by dermal fibroblasts (60). This has been clearly demonstrated in the Ouwehand et al. study, where the migration of LC into the dermis was abrogated by CXCR4 and CXCL12 blocking antibodies (60). CCR7 did play a role in the migration of LCs from the epidermis to the dermal layer as the blocking of CCR7 ligands CCL19 and CCL21 did not affect the migration (60). To summarize, the migration of LCs into the lymph node has two phases, after the initial exposure to inflammatory stimuli such as hapten, LCs migrate into the dermis in a CXCR4–CXCL12-dependent manner but not reliant on CCR7. Once LCs have resided in the dermis, they upregulate their expression of CCR7, enter the lymphatic system, and migrate into the lymph node. These additional steps could be the reason for the delayed migration of LCs into the lymph node in comparison to dermal DCs. The CXCR4–CXCL12 axis governs LC migration to the LN in both mice and human, as shown in both studies (59, 60).

Although migration of dermal DCs and LCs has been well described by multiple studies, it has been challenging to accurately quantify the movement and lifespan of skin-derived DC subsets and LCs. The use of Kaede-transgenic mice has allowed researchers to investigate the exact kinetics of the migration of DCs and LCs into the dLNs. Kaede-transgenic mice express a green-to-red photoconvertible protein, Kaede (61). More recently, the use of KikGR mice has been used to track immune cell movement from the skin to the dLN after photoconversion. Similar to Kaede mice, KikGR fluorescence changes irreversibly from green to red upon exposure to violet light; as a result, KikGR mice have a greater photoconversion efficiency as compared to Kaede mice (62, 63). Tomura et al. were able to show the kinetics of LCs, CD103^+^ dermal DC, and CD103^−^ into the dLN (64). After irritation, the number of LCs in dLN only peak from day 4 onward, as compared to CD103^+^ and CD103^−^ dermal DCs, which migrate to dLN almost immediately after irritation. Additionally, only photoconverted LCs migrated into the dLN, emphasizing the fact that LCs do not get replaced by blood-supplied precursors during steady state (25, 64).

Understanding the kinetics and mechanisms of LCs migration into the dLN is crucial as there is clear evidence suggesting that the skin is one of the main pathways that leads to developing systemic immunity by exogenous protein antigens (65). LCs have shown to have slower migration to the dLN as compared to the dermal DCs, although the consequence of slower migration by antigen-presenting cells has yet to be evaluated. Additionally, in these kinetics studies, the possible role of IDEC and LC-like cells’ migration into the dLN has yet to be discussed and studied. As we understand, IDECs and LC-like cells have very similar expression and phenotype as true resident LCs; therefore, it is difficult to evaluate if resident LC is solely responsible for antigen presentation in the dLN from the epidermis.

## Conclusion

Langerhans cells are unique cells residing in the upper layer of the epidermis and are now regarded as part of the macrophage family due to its developmental pathway and ontogeny. LCs, as many other tissue-resident macrophages, arise from embryonic precursors and are maintained in the epidermis by low levels of proliferation and self-renewal in steady-state conditions. Unlike many other tissue-resident macrophages, LCs are the only macrophage that possess both DC and macrophage properties. They arise from embryonic precursors and survey antigens on the epidermal surface (properties, which are characteristic of macrophages); yet are able to migrate into the dLN, which provides evidence that they share a functional blueprint with DCs. The dual identity of LCs substantiates the plasticity of LCs, although the molecular mechanisms and developmental pathways of a *bona fide* LC in the epidermis have yet to be clarified. LCs have also shown to have the capability to adapt to different immune stimuli in the skin, both in the areas of immunosuppression and in the pro-inflammatory responses. However, the presence of IDECs and LC-like cells coupled with the lack of identification and classification between these three cell types could have led to mischaracterization of the role of LCs during inflammation in past studies. Therefore, there is a need to explore the role of IDECs and LC-like cells in order to truly understand the role of LCs particularly during inflammation. This can lead to the discovery of novel immune modulators, which can be used for the treatment of inflammatory skin diseases.

## Author Contributions

MO wrote the mini review and contributed to the intellectual input and the structure of the review, GE and KK supervised the writing and the direction of the mini review, and GE and KK also contributed to the intellectual input and edited the mini review.

## Conflict of Interest Statement

The authors declare that the research was conducted in the absence of any commercial or financial relationships that could be construed as a potential conflict of interest.
